# Stochastic and multi-objective design of photonic devices with machine learning

**DOI:** 10.1038/s41598-024-57315-4

**Published:** 2024-03-26

**Authors:** Paolo Manfredi, Abi Waqas, Daniele Melati

**Affiliations:** 1 Department of Electronics and Telecommunications, Politecnico di Torino, 10129 Turin, Italy; 2 Department of Telecommunication, Mehran University of Engineering and Technology, Jamshoro, Pakistan; 3 Center for Nanoscience and Nanotechnologies, CNRS, Université Paris-Saclay, 10 Bv. Thomas Gobert, 91120 Palaiseau, France; 4 Now at Tyndall National Institute, Lee Maltings, University College Cork, T12 R5CP Cork, Ireland

**Keywords:** Integrated optics, Silicon photonics

## Abstract

Compact and highly performing photonic devices are characterized by non-intuitive geometries, a large number of parameters, and multiple figures of merit. Optimization and machine learning techniques have been explored to handle these complex designs, but the existing approaches often overlook stochastic quantities. As an example, random fabrication uncertainties critically determines experimental device performance. Here, we present a novel approach for the stochastic multi-objective design of photonic devices combining unsupervised dimensionality reduction and Gaussian process regression. The proposed approach allows to efficiently identify promising alternative designs and model the statistic of their response. Incorporating both deterministic and stochastic quantities into the design process enables a comprehensive analysis of the device and of the possible trade-offs between different performance metrics. As a proof-of-concept, we investigate surface gratings for fiber coupling in a silicon-on-insulator platform, considering variability in structure sizes, silicon thickness, and multi-step etch alignment. We analyze 86 alternative designs presenting comparable performance when neglecting variability, discovering on the contrary marked differences in yield and worst-case figures for both fiber coupling efficiency and back-reflections. Pareto frontiers demonstrating optimized device robustness are identified as well, offering a powerful tool for the design and optimization of photonic devices with stochastic figures of merit.

## Introduction

Innovative photonic devices and systems are at the base of many transformative technologies, such as high-speed optical communication and computing, ultrasensitive biochemical detection, super-resolution imaging, and quantum information processing. These advancements demand for photonic components achieving simultaneously a large scale of integration and high performance^1^, leading to ever more complex designs characterized by a large number of geometrical and material parameters. At the same time, modern cutting-edge designs usually involve multiple figures of merit that account for both performance metrics and fabrication requirements, thus complicating the selection of the final design candidates and requiring multi-objective analysis and optimization tools.

Recently, researchers have proposed inverse design methods to efficiently explore the vast design space of multi-parameter photonic devices and possibly take into account multiple figures of merit^2–6^. Inverse design algorithms are essentially rule-based approaches that use iterative searching steps on a case-by-case basis, often relying on numerical simulations in each step to produce intermediate results that help modify the search strategy. To this purpose, several optimization algorithms have been proposed and tested, including heuristic methods, such as genetic algorithms and particle swarm, and gradient-based ones. These approaches help discover non-intuitive photonic structures that outperform in compactness and, recently, also performance those obtained relying on the experience and physics intuition of the designer. Machine learning algorithms have been demonstrated to empower and speed up the design process by creating models capable of inexpensively predicting the optical response of a structure, directly solving the inverse design problem, or reducing the dimensionality of the design space^7–12^.

However, the approaches proposed so far for multi-parameter and multi-objective design often focus only on deterministic figures of merit, such as the ideal efficiency or the bandwidth of a device. On the other hand, stochastic quantities play an ever-growing and critical role in high-performance devices and must be taken into account in the design process. The most striking example is represented by the impact of fabrication imperfections. Dimensional variations are unavoidable, limit the sustainable complexity of circuits, and pose significant challenges in achieving high fabrication yield. This is particularly true for high-index-contrast technologies, where minor fabrication deviations in waveguide geometry and circuit topology have a large impact on light propagation and device response^13–15^. To address this problem, a possible approach is to quantify the impact of uncertainty on the device performance and optimize the design to ensure a robust behavior against fabrication tolerances^16^. In the multi-parameter, multi-objective scenario considered here, Monte Carlo analysis is not a viable solution due to its computational inefficiency combined with the enormous space of fabricable devices. Indeed, in the context of design exploration and optimization, each stochastic figure of merit would need to be re-evaluated for each parameter configuration. This would require millions of simulations, thereby making the problem computationally prohibitive.

In order to overcome these limitations, several modelling approaches were investigated to surrogate computationally expensive systems and accelerate iterative simulations^17,18^. In particular, stochastic spectral methods based on the generalised polynomial chaos have emerged as a promising alternative, significantly outperforming Monte Carlo. Sparse implementations (e.g., least-angle regression, sparse interpolations, and low-rank tensor decompositions) are also appropriate for high-dimensional problems^19–22^. All these techniques, including the sparse ones, are however parametric, meaning that the form of the predictor must be specified beforehand. This is a critical limitation for problems exhibiting a design space with large variability, since such parametric models often do not generalize well. Moreover, their complexity is directly proportional to the number of input design variables.

In this regard, an effective alternative is the class of nonparametric machine learning methods, for which the model complexity is not related to the problem dimensionality, but rather to the number of available training data^19,23–25^. One example is Gaussian process regression (GPR)^26^, also known as Kriging^27^. An advantage of nonparametric models is that they are purely data-driven, and therefore they can adapt better to the analysis of complex devices compared to other methods, like polynomial chaos, that assume a predefined model form. Furthermore, the nonparametric nature of these methods makes them even more appealing for high-dimensional problems. For example, enhanced variants of GPR have been proposed to address the “curse of dimensionality” for systems with a large number of inputs^19^. GPR assumes that the target function is a realization from a Gaussian process and uses Bayesian inference, conditioned on a limited number of observed data, to identify it. In fact, the model is probabilistic in nature, and the model output for a given input can be interpreted as the most likely prediction over the possible Gaussian process realizations. In contrast to other machine learning methods such as neural networks, GPR and other Bayesian methods offer the advantage of being rather parsimonious in terms of training data^28^.

Here, we propose a new approach based on machine learning to include stochastic figures of merit in the multi-objective design of photonic devices characterized by multiple parameters. In particular, we combine for the first time unsupervised dimensionality reduction with GPR. The use of dimensionality reduction allows representing different device designs using a smaller number of parameters compared to the original design space. Within this lower-dimensional design sub-space, we efficiently sample tens of alternative designs and build GPR surrogates to accurately model their response to parameter variability with a minimal computational effort. In this way, it becomes possible to map an arbitrary number of stochastic figures of merit over the entire design sub-space, highlighting strong differences in robustness to uncertainty and enabling the multi-objective optimization of the device. As a proof-of-concept, we analyse surface gratings for fiber coupling in a silicon-on-insulator (SOI) platform subject to multiple sources of parameter variability (i.e., width and thickness deviations and alignment of multiple etch steps). We compute worst case and yield performances for tens of different designs considering uncertainty of both fiber coupling efficiency and back-reflections, and we demonstrate the existence of Pareto frontiers optimizing device robustness against different metrics.

## Results

### Multi-objective stochastic analysis

In this work, we consider photonic devices characterized by a relatively large number of design parameters $\varvec{x} = \{x_1, ..., x_T\}$ and for whom multiple figures of merit must be considered simultaneously (multi-objective analysis). The figures of merit include both deterministic quantities $\varvec{F} = \{F_1,..., F_N\}$ and stochastic quantities $\varvec{p} = \{p_1,..., p_K\}$ that result from parameters variability.

**Figure 1:**
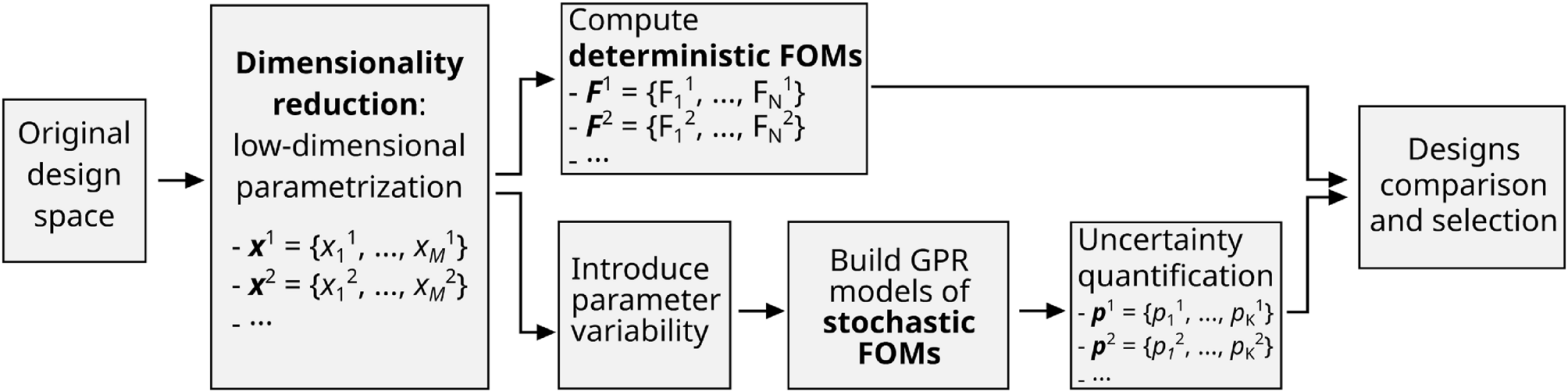
Overview of the proposed approach for the multi-objective analysis and optimization of photonic devices including stochastic figures of merit. FOMs, figures of merits. GPR Gaussian process regression.

The approach we propose for the analysis and multi-objective optimization of such devices extends the framework proposed in Ref.^9^ and is schematically represented in Fig. 1. As detailed in the Methods section, we rely on the use of dimensionality reduction to analyze the relationship between the parameters of the device in the original highly-dimensional design space and identify a lower-dimensional parametrization with minimal loss of information. We exploit in particular Principal Component Analysis. Since dimensionality reduction largely reduces the number of parameters required to describe a device from T to M, with $M\ll T$, it becomes possible to sweep them and compute any required figure of merit for all parameter combinations.

While deterministic quantities can be readily simulated, in order to include stochastic quantities an efficient computational method is fundamental and to this purpose we introduce the use of GPR surrogates. GPR assumes the target function to be a particular realization of a Gaussian process, which is called *prior*. The prior is characterized by a mean function, or *trend*, $\mu (\varvec{x}):\mathbb {R}^M\rightarrow \mathbb {R}$ and by a covariance function, or *kernel*, $k(\varvec{x},\varvec{x}^\prime ):\mathbb {R}^M\times \mathbb {R}^M\rightarrow \mathbb {R}$. The trend is a function of the design parameters $\varvec{x}$ and embeds a possible prior belief on the general behaviour of the target function w.r.t. such parameters. It is usually described as a linear combination of predefined basis functions (e.g. polynomials up to a given order) with coefficients that are determined as part of the training process. A constant or even zero trend can be used when such information is not available. The kernel is a function of a pair $(\varvec{x},\varvec{x}^\prime )$ of design parameters or, more frequently, of their distance $\left\Vert \varvec{x}-\varvec{x}^\prime \right\Vert$ in the design space (in which case the kernel is said to be “stationary”). The kernel describes how much and, especially, how smoothly the target function varies w.r.t. the design parameters. Commonly, a particular form of kernel function is selected a priori (popular choices are the squared-exponential or Matérn kernels), and then pertinent coefficients thereof are estimated as part of the training process. In order to train the GPR model, observations are collected from the target function (i.e., from the actual simulation model) and Bayesian inference is used to identify the process realization that best fits to the data. The theory of conditional probability is used to find the “trajectory” that is more consistent with the observed data. A crucial point is thus to choose a good prior for the problem at hand. However, one of the interesting properties of GPR models is that they exhibit a large amount of flexibility and adaptability. Indeed, one typically needs only to make some mild assumption on the data (e.g., relative smoothness, periodicity, etc.), select a reasonable trend and covariance model, and then optimize the “degrees of freedom” (the trend coefficients and kernel parameters) based on the observations in order to adapt them to the specific problem at hand.

### Problem setup: vertical surface grating coupler

**Figure 2:**
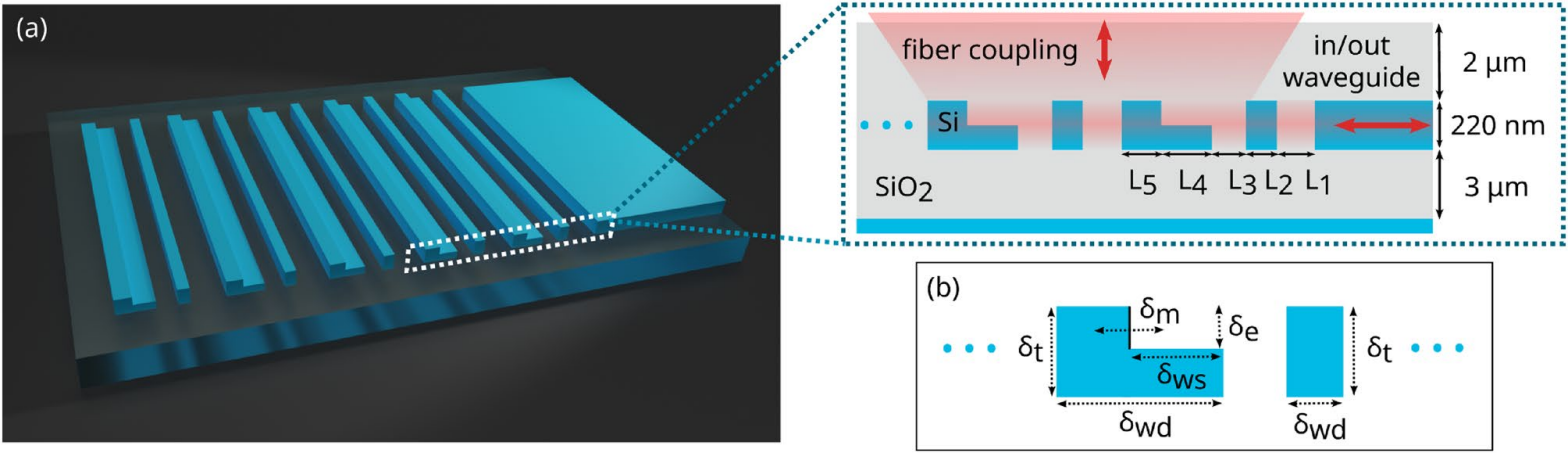
Surface grating for fiber-chip coupling. He we consider a fiber placed perfectly vertical on top of the chip. (**a**) 3D schematic view of the device, realized on a standard, 220-nm SOI platform with a double etching process. Each unit cell of the periodic structure includes five different sections of length L_1_ - L_5_. (**b**) Fabrication variability considered in the analysis: thickness of the silicon layer ($\mathrm {\delta }_{t}$); width of the deeply etched structures ($\mathrm {\delta }_{wd}$); width of the partially etched areas ($\mathrm {\delta }_{ws}$) and their etch depth ($\mathrm {\delta }_{e}$); alignment between partially and fully etched areas ($\mathrm {\delta }_{m}$), resulting in a displacement of the partially etched wall within the L-shaped geometry (marked by a solid black line).

As a case study, we apply the methodology described in the previous section to the analysis and optimization of a surface grating designed to couple light between an integrated waveguide and a standard optical fiber placed orthogonally on top of the chip. The design of surface gratings with a perfectly vertical emission is known to be a challenging problem because this condition results in the appearance of a second diffraction order whose excitation must be suppressed to avoid a large part of the optical power to be reflected into the input waveguide^29^. A multi-objective approach to the design is hence crucial even in the case of an “ideal” device without parameter variability, since fiber-chip coupling efficiency and back-reflections have to be taken simultaneously into account as figures of merit.

We consider here the structure schematically represented in Fig. 2a^9,30^. The device is designed in a standard SOI platform and each unit cell of the periodic grating consists of a pillar of 220 nm in height and an L-shaped section with a partial etch to 110 nm. Each of the five sections in the unit cell has a length L_i_, and the grating period is hence $\Lambda = \sum _{i=1}^5 \text{L}_i$.

**Figure 3:**
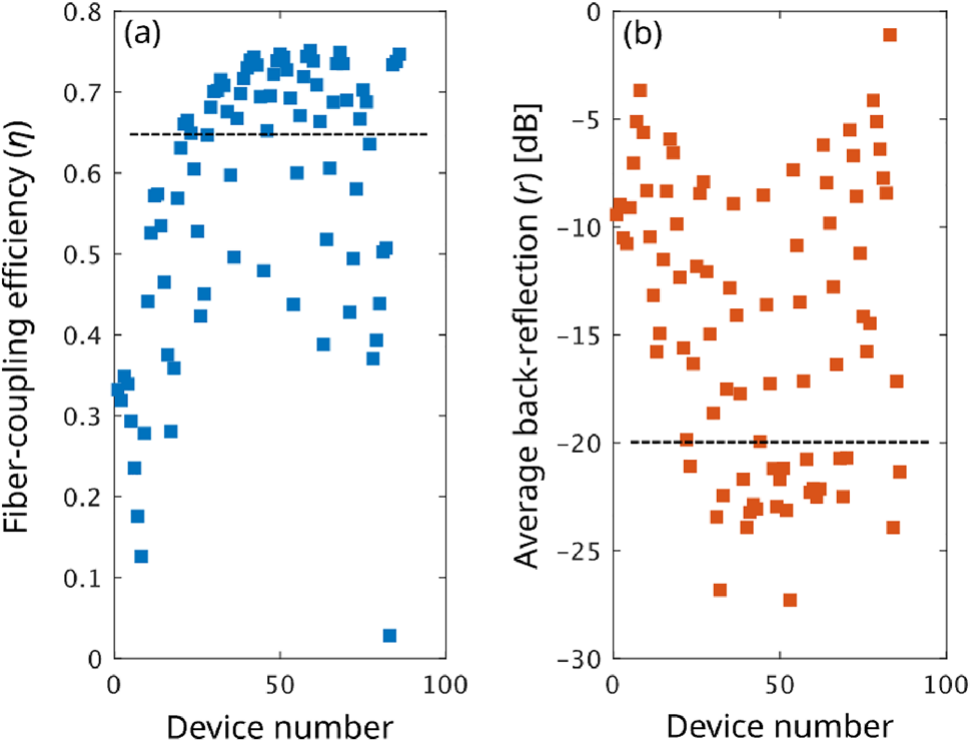
(**a**) Fiber-chip coupling efficiency $\eta$ at $\lambda =1550$ nm for the initial collections of 86 alternative designs and (**b**) the corresponding back-reflection *r*, averaged over the optical communication C band (1530 nm – 1565 nm). The dashed lines mark $\eta =0.65$ and $r=-20$ dB, respectively.

The original design space of the grating is five-dimensional (defined by the five section lengths $\varvec{L}$ = {L_1_, ..., L_5_}). As a result of dimensionality reduction through Principal Component Analysis, the parameters are reduced to two effective parameters that are then swept, sampling 86 possible alternative designs. For all of them, we compute both the fiber-chip coupling efficiency $\eta$ and back-reflections *r* in the input waveguide, which are reported in Fig. 3a and b, respectively. In this work, we define *r* as the average grating back-reflection over the optical communication C band (1530 nm – 1565 nm), an approach that is more realistic than considering reflections at a single wavelength. Coupling efficiency $\eta$ is instead evaluated at $\lambda _0$ = 1550 nm, the required operative wavelength. The set of generated designs includes 24 high-performing gratings with $\eta >0.65$ and/or $r<-20$ dB (threshold are marked by dashed lines in Fig. 3a and b).

Uncertainty is then introduced as parameter fluctuations generated by fabrication imperfections. We consider in particular a complex uncertainty scenario with five different random variables, represented in Fig. 2b: variations of the thickness of the silicon layer ($\mathrm {\delta }_{t}$, with standard deviation $\sigma$ = 3 nm); variations of the width of both the deeply etched ($\mathrm {\delta }_{wd}$, $\sigma$ = 5 nm) and partially etched ($\mathrm {\delta }_{ws}$, $\sigma$ = 5 nm) structures caused by lithography and etching; a limited control of the etch depth of the partially etched areas ($\mathrm {\delta }_{e}$, $\sigma$ = 5 nm); alignment tolerance between the partially and fully etched areas ($\mathrm {\delta }_{m}$, $\sigma$ = 10 nm), which results in a variation of the aspect ratio of the L-shaped geometry. All the variables are independent and Gaussian-distributed, with zero mean and the standard deviations marked above The described uncertainty model represents a realistic fabrication process for SOI devices^31,32^ and could be easily adapted to match the platform characteristics of a specific foundry.

Beside the two mentioned deterministic quantities ($\eta$ and *r*), we introduce four additional stochastic figures of merit, again related to efficiency and back-reflections, and based either on quantiles or on probability. In particular, the quantile-based indicators are defined as the 10% quantile of the efficiency at the operating wavelength $\lambda _0$, i.e.,

1 $$\begin{aligned} q_\eta =\left\{ x:P[\eta <x]=10\%\right\} \equiv \left\{ x:P[\eta >x]=90\%\right\} \end{aligned}$$

and the 90% quantile of the average back reflection *r*, i.e.,

2 $$\begin{aligned} q_r=\left\{ x:P[r<x]=90\%\right\}. \end{aligned}$$

Therefore, $q_\eta$ ($q_r$) represents the minimum (maximum) value of efficiency (average back reflection) above which (below which) we find 90% of the design samples. Hence, it can be thought of as a sort of “probabilistic worst-case” indicator. The percentage-based indicators are instead defined as

3 $$\begin{aligned} p_\eta =P[\eta >\gamma _\eta ] \end{aligned}$$

and

4 $$\begin{aligned} p_r=P[r<\gamma _r] \end{aligned}$$

for the efficiency and back-reflection, respectively, where $\gamma _\eta =0.65$ and $\gamma _r=-20$ dB are target values that are representative of an acceptable design. Therefore, they can be considered as yield indicators.

### Preliminary validation of the approach

Before exploiting the proposed approach in the multi-objective analysis and optimization of the full batch of 86 available designs, we first validate the method using only the last three designs in the batch, characterized by the following nominal lengths:

- 84: $$\varvec{L}^{84}=\{77, 84, 115, 249, 171\}$$ nm;
- 85: $$\varvec{L}^{85}=\{102, 80, 117, 329, 97\}$$ nm;
- 86: $$\varvec{L}^{86}=\{84, 84, 110, 284, 142\}$$ nm.

These designs have a fiber coupling efficiency of 0.73, 0.74, and 0.75, and average back-reflection of $-24$ dB, $-17$ dB, and $-21$ dB, respectively. For each of the three designs, 1100 Monte Carlo simulations are performed for randomly drawn configurations of the aforementioned five uncertain parameters. We use a subset of 100 samples to train the GPR models and the remaining 1000 samples for validating the model accuracy.

**Figure 4:**
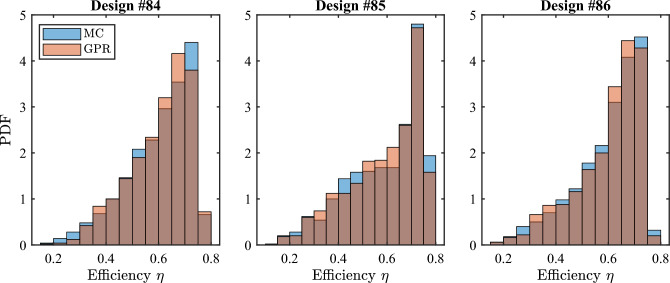
Probability distribution of the coupling efficiency $\eta$ at the central wavelength $\lambda$ = 1550 nm for designs #84, #85, and #86 when fabrication uncertainty is considered. Blue histogram: reference distribution from the Monte Carlo samples; red bars: distribution predicted by means of the GPR model in conjunction with PCA compression.

**Figure 5:**
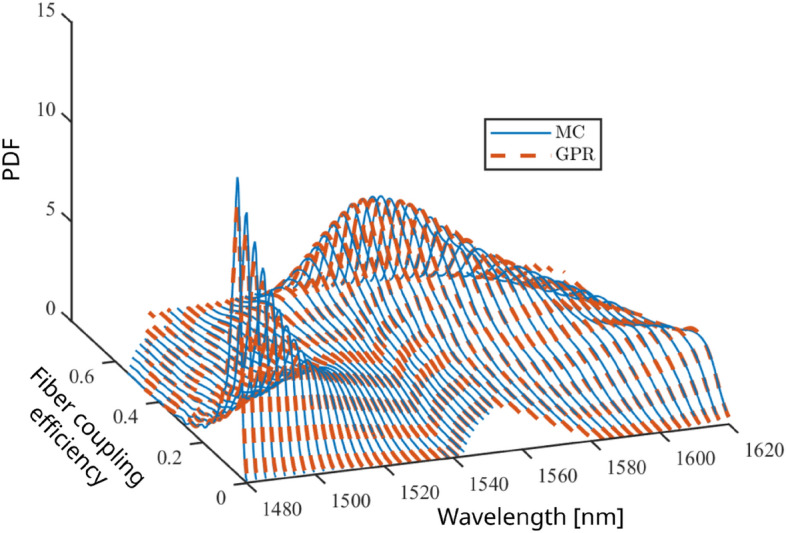
Probability distribution of the coupling efficiency $\eta$ of design #86 as a function of the wavelength. Solid blue lines: reference distribution from the Monte Carlo samples; dashed red lines: distribution predicted with the GPR model.

As a first validation, we consider the coupling efficiency $\eta$ of three selected designs at the central wavelength $\lambda _0$. In Fig. 4, the probability density function (PDF) of the efficiency predicted by the GPR models (red histogram) is compared against the reference distribution of the Monte Carlo samples (blue bars). An excellent agreement is established in all the three cases. Next, we focus on the efficiency of design #86 as a function of the wavelength $\lambda$. Figure 5 shows the PDF at 51 wavelengths between 1480 nm and 1620 nm. The distributions of the Monte Carlo samples (solid blue lines) are compared against the corresponding predictions obtained with the GPR model (dashed red lines), highlighting again a remarkable accuracy.

To obtain the above results, PCA is adopted to compress the wavelength-dependent data and reduce the number of separate models to be trained for each individual design, thereby improving the training efficiency^19^. By setting a 0.1% relative threshold on the singular values, the number of retained principal components (and hence, of separate models to be trained) is 18, 17, and 18 for designs #84, #85, and #86, respectively. It should be noted that the training phase of the GPR models requires about 3 seconds for each design, whereas the validation, i.e., the model evaluation for the remaining 1000 samples not considered for training, takes less than 1 second. The above computational times are negligible compared to the Monte Carlo runs (about 2 minutes per simulation).

**Figure 6:**
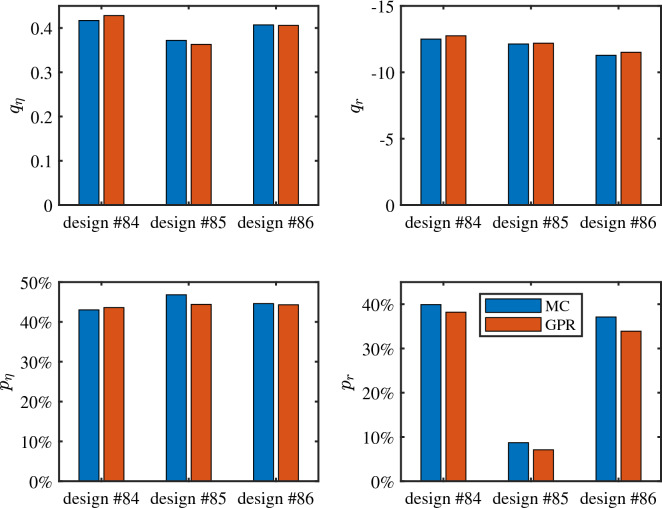
Comparison between worst case and yield indicators computed from the Monte Carlo samples (blue bars) and predicted by the GPR models (red bars).

Next, we compute the four indicators described in the previous section for the three validation designs. Results are reported in Fig. 6. Results from the Monte Carlo analysis (blue bars) compare well with the GPR predictions (red bars). The latter are obtained by directly training two separate models for the efficiency at $\lambda _0$ = 1550 nm and for the average back-reflection over the optical communication C band, without the use of PCA. From Fig. 6, it is possible to draw some interesting conclusions. The most striking result is the much lower value of $p_r$ for design #85, whereas $q_r$ is similar for all designs. This means that all the three designs perform similarly in terms of worst-case back-reflection, with a maximum value within $[-12.5, -11.3]$ dB for most samples (i.e., 90%). However, for design #85, less than 10% of the samples achieve an average back reflection below the target value of $-20$ dB. The amount is much higher for design #84 and #86 (40% and 37%, respectively), which have therefore a similar and much higher yield compared to design #85. On the other hand, design #85 exhibits the best yield in terms of efficiency (47% of the samples meet the target specification), but also the lowest worst-case efficiency. This analysis highlights that the two figures of merit (efficiency and back reflection) are potentially competing, and it is therefore important to find a design that is sufficiently good in both metrics.

### Optimization results

After the successful validation of the previous section, the GPR surrogates are used for the exploration of all the 86 alternative designs. The same prior type is used as for the three validation designs. For each design, only 100 training samples are generated using a Monte Carlo analysis. A much larger number of 10000 samples is then inexpensively generated using the trained GPR surrogates to accurately evaluate the aforementioned stochastic performance metrics.

**Figure 7:**
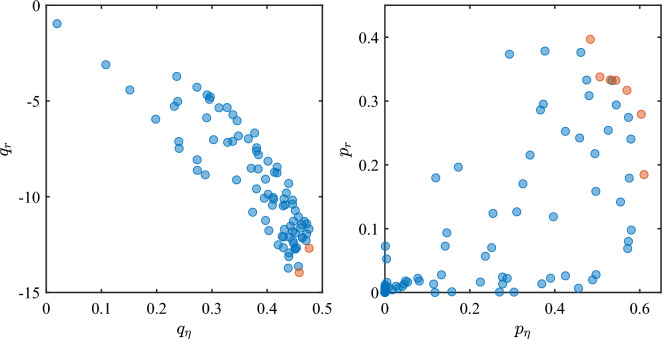
Scatter plots pairing worst case (left) and yield (right) performance indicators for each design. Red circles indicate designs that belong to the Pareto front.

Figure 7 shows the scatter plots of the $(q_\eta ,q_r)$ and $(p_\eta ,p_r)$ indicator pairs. The red dots indicate the Pareto front of the designs. A design belongs to the Pareto front if there are no other designs that dominate it (i.e., are better than it) in all metrics. As seen from Fig. 7, there is no design that simultaneously performs better than any other design in both metrics. In particular, there are two Pareto-optimal designs as far as the quantile-based (worst-case) indicators are concerned:

- 52: $\varvec{L}^{52}=\{62, 93, 79, 282, 159\}$ nm, $q_\eta =0.476$, $q_r=-12.7$ dB, $\eta =0.73$, $r=-23$ dB;
- 53: $\varvec{L}^{53}=\{50, 98, 60, 280, 170\}$ nm, $q_\eta =0.458$, $q_r=-14.0$ dB, $\eta =0.70$, $r=-27$ dB.

There are instead seven Pareto-optimal designs w.r.t. the probability-based (yield) indicators:

- 42: $$\varvec{L}^{42}=\{76, 86, 108, 262, 163\}$$ nm, $$p_\eta =53.1\%$$, $$p_r=33.3\%$$, $$\eta =0.74$$, $$r=-23$$ dB;
- 43: $$\varvec{L}^{43}=\{63, 91, 89, 260, 174\}$$ nm, $$p_\eta =48.4\%$$, $$p_r=39.7\%$$, $$\eta =0.73$$, $$r=-23$$ dB;
- 51: $$\varvec{L}^{51}=\{76, 88, 97, 284, 148\}$$ nm, $$p_\eta =54.4\%$$, $$p_r=33.3\%$$, $$\eta =0.74$$, $$r=-21$$ dB;
- 58: $$\varvec{L}^{58}=\{101, 79, 125, 310, 111\}$$ nm, $$p_\eta =61.0\%$$, $$p_r=18.5\%$$, $$\eta =0.74$$, $$r=-21$$ dB;
- 59: $$\varvec{L}^{59}=\{88, 84, 106, 308, 122\}$$ nm, $$p_\eta =60.4\%$$, $$p_r=27.9\%$$, $$\eta =0.75$$, $$r=-22$$ dB;
- 84: $$\varvec{L}^{84}=\{77, 84, 115, 249, 171\}$$ nm, $$p_\eta =50.6\%$$, $$p_r=33.8\%$$, $$\eta =0.73$$, $$r=-24$$ dB;
- 86: $$\varvec{L}^{86}=\{84, 84, 110, 284, 142\}$$ nm, $$p_\eta =57.0\%$$, $$p_r=31.7\%$$, $$\eta =0.74$$, $$r=-21$$ dB.

**Figure 8:**
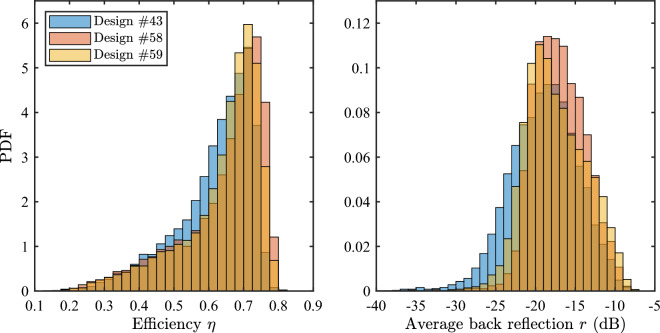
PDF of the efficiency (left) and average back reflection (right) of three selected designs beloning to the Pareto front of the yield indicators.

Ideal fiber coupling efficiency and back reflection (computed without considering parameter variability) are reported alongside with the stochastic figures of merit. By definition of the Pareto front, the designs that exhibit the best performance in one metric have the lowest performance in the other metric. It is also worth noting that no design belongs to the Pareto front of both the worst-case and yield indicators. However, the designer can focus on the Pareto-optimal designs to make further considerations and find a trade-off. For example, design #59 has a yield on the efficiency that is only 0.6% worse than the best one, provided by design #58. However, it exhibits a much larger yield on back reflection, thus being a good candidate to be considered as the overall optimum. The PDF of both figures of merit for designs #58 and #59 are shown in Fig. 8 (red and yellow histograms, respectively). The distributions for design #43, i.e., the best in terms of back reflection yield, is also included (blue histogram). It is observed that, consistently with the previous conclusions, the back reflection distribution for design #43 is the one shifted to the lowest values. However, this applies also to the efficiency distribution, indicating a worse performance in that metric. Moreover, the efficiency distributions for design #58 and #59 are confirmed to be very similar. Nevertheless, the back reflection distribution for design #59 is visibly shifted towards lower values, indicating a substantially better performance in that metric, as noticed before.

Finally, it is interesting to notice that the higher yield for design #59 in terms of back reflection is achieved despite the ideal value being almost identical to that of design #58. Likewise, design #43 has the best back-reflection yield in the dataset but not the best ideal back reflection (which belongs to design #53, with $-27$ dB). More generally, for all the designs in the Pareto front of the yield, ideal coupling efficiency and back reflection only show negligible fluctuations. On the contrary, marked differences can be seen in both yield metrics, with variations of more than 12% and 15%, respectively.

## Discussion and conclusions

We have proposed a new approach for the multi-objective analysis and optimization of photonic devices characterized by multiple parameters and stochastic figures of merit. Our methodology relies on dimensionality reduction to efficiently sample alternative designs with high ideal performances (i.e., computed without parameter variability) and on the use of GPR surrogates to accurately model their response to uncertainty. Single-objective optimization techniques commonly provide a single design solution with little to no information about achievable performance and possible trade-offs. The availability of a pool of alternative designs with different characteristics, such as that provided by multi-objective approaches, helps the designer gaining a global perspective of the device behaviour, revealing performance and structural limitations, and possibly inspiring new design approaches^9,33^. Moreover, additional figures of merit can be calculated for the solutions already available in the pool at any stage of the design process, enriching the analysis without having to restart the entire optimization from scratch. Within this framework, an efficient methodology for the computation of stochastic quantities becomes critical. Even with a low-dimensional parameterization, the sampling of the design space may include tens or hundreds of alternative designs and each of them may require the calculation of several figures of merit, making Monte Carlo methods unfeasible.

On the contrary, the approach proposed here made it possible to compute the stochastic behaviour of coupling efficiency and back-reflection for 86 designs of a vertical grating coupler using a mere 100 training samples, compared to the (several) thousands required by Monte Carlo. We identified Pareto frontiers based on worst-case and yield indicators, highlighting significant differences among the alternative designs and the (competing) balance between the different figures of merit. Moreover, we showed that designs with the same ideal performances can have striking differences in terms of robustness to uncertainty, demonstrating the importance of including stochastic figures of merit as part of the multi-objective design of highly performing photonic devices.

## Methods

### Grating coupler simulation

The simulation of coupling efficiency and back reflection for each design of the grating coupler is performed by means of a commercial 2D-FDTD solver. The waveguide structure includes a silicon substrate, 2-$\mathrm {\mu {}}$m buried oxide, 220nm-thick silicon core, and a silica upper cladding of 1.5 $\mathrm {\mu {}}$m thickness. Silicon and silica refractive indices are 3.45 and 1.45 at $\lambda$ = 1550 nm. The mode of an SMF-28 single-mode optical fiber is modeled as a Gaussian function with a mode field diameter of 10.4 $\mathrm {\mu {}}$m. The fiber facet is assumed to be in direct contact with the top of the upper cladding and its longitudinal position along the grating is optimized for each design to maximize the coupling efficiency. Transverse-electric (TE) polarized light is injected through an input optical waveguide and the fiber coupling efficiency is calculated as the overlap integral between the simulated field diffracted upwards by the grating and the Gaussian function. Back reflections are computed as the fraction of the optical power coupled to the counter-propagating TE mode of the input waveguide.

### Dimensionality reduction for device design

The methodological framework exploited here relies on three main steps to efficiently address the design of photonic devices characterized by many design parameters and enable the efficient computation of multiple figures of merit. In the first stage, multiple iterations of a local optimization algorithm are used to generate a sparse collection of different “good” designs, i.e., designs that optimize one (deterministic) performance criterion that is chosen as the essential and most prominent one (e.g., the ideal efficiency). Each iteration of the optimizer is initialized either with a random guess or with a physics-informed set of parameters. For the design examples described in this work, we used in particular a custom-made line search algorithm. In the second stage, machine learning dimensionality reduction is applied to analyze the relationship in the parameter space between these degenerate designs. The goal is to find a lower-dimensional sub-space that contains all good designs. The advantage is that this design sub-space is described by significantly fewer parameters compared with the original design space. For the grating coupler example, we used linear Principal Component Analysis as the dimensionality reduction method. Five initial good designs with fiber coupling efficiency larger than 0.74 were used to compress the design space to two hyper-parameters. In the last stage, we efficiently sample the design sub-space and create a collection of alternative device designs. Because of the construction method, we are guaranteed that a large fraction of these alternative designs are potentially of interest, in the sense that they optimize at least the most important performance metric. Any additional figure of merit can be computed within the sub-space, readily introducing multi-objective analysis and optimization capabilities.

### Gaussian process regression

In order to train the GPR model, we collect *L* observations $\{y_l\}_{l=1}^L$ of the desired output quantity, computed for as many configurations $\{\varvec{x}_l\}_{l=1}^L$ of the input design parameters. The input configurations are randomly drawn according to their probability distribution. In the considered simulations, the output quantities of interest are, e.g., the efficiency $\eta$ at the central wavelength, the average back reflection *r* in the C band, or the principal components of the wavelength-dependent metrics. We choose to use a simple linear model for the Gaussian process trend, i.e.,

5 $$\begin{aligned} \mu (\varvec{x})=\beta _0+\sum _{m=1}^M\beta _mx_m \end{aligned}$$

and an anisotropic Matérn 5/2 kernel for the covariance function, i.e.,

6 $$\begin{aligned} k(\varvec{x},\varvec{x}^\prime )=\sigma ^2\left( 1+\sqrt{5}r+\frac{5}{3}r^2\right) \exp \left( -\sqrt{5}r\right) , \end{aligned}$$

with

7 $$\begin{aligned} r=\sqrt{\sum _{m=1}^M\frac{(x_m-x^\prime _m)^2}{\theta _m^2}}, \end{aligned}$$

which is one of the most popular due to its excellent generalization properties. This is a stationary kernel, since the covariance value depends only on the distance between the points, regardless of their absolute value. The term “anisotropic” refers to the fact that a different smoothness parameter $\theta _m$ (lengthscale) is used for each input dimension. This further improves the adaptability and the accuracy of the model, since the function is allowed responding with different smoothness to each design parameter. The vector of trend coefficients $\varvec{\beta }=(\beta _0,\ldots ,\beta _M)$ is computed by means of a generalized least-square regression, whereas the kernel variance $\sigma ^2$ and lengthscales $\varvec{\theta }=(\theta _1,\ldots ,\theta _M)$ (the so-called “hyperparameters” of the GPR model) are obtained via a maximum likelihood estimation. Hence, the parameters are selected such that they maximize the likelihood that the data comes for the corresponding process. It should be noted that the type of trend and kernel could be optimized as well, starting from a predefined pool of candidates. However, this choice is discarded as it considerably increases the training time while leading to marginal accuracy improvements.

Once the prior parameters $(\varvec{\beta },\sigma ^2,\varvec{\theta })$ are estimated, the GPR model prediction at a generic point $\varvec{x}^*$ is given by the expectation of the posterior process, i.e., the process that is conditioned on the observed data, leading to^34^

8 $$\begin{aligned} y\approx \mathcal {M}_\text{GPR}(\varvec{x}^*)=\varvec{h}(\varvec{x}^*)^T\varvec{\beta } +\varvec{r}(\varvec{x}^*)^T\textbf{R}^{-1}(\varvec{y}-\textbf{H}\varvec{\beta }) \end{aligned}$$

where

- $\varvec{y}=(y_1,\ldots ,y_L)^T$ is the vector of training observations;
- $\textbf{R}$ is the $L\times L$ correlation matrix of the training samples, with $R_{lk}=k(\varvec{x}_l,\varvec{x}_k)/\sigma ^2$, $l,k=1,\ldots ,L$;
- $\textbf{H}$ is the $L\times (M+1)$ matrix of the trend regressors evaluated at the training samples, i.e., the *l*-th row of $\textbf{H}$ is the vector $(1,x_{l1},\ldots ,x_{lM})$;
- $\varvec{r}$ is the cross-correlation vector between the prediction point and the training samples, i.e., $r_l=k(\varvec{x}^*,\varvec{x}_l)/\sigma ^2$;
- $\varvec{h}$ is the vector of trend regressors evaluated at the prediction point, i.e., $\varvec{h}=(1,x_1^*,\ldots ,x_M^*)$.

It should be noted that the model prediction does not depend on the kernel variance $\sigma ^2$. However, this information can be used to assess the confidence of the predictions^19,26,34^.

### Principal component analysis for wavelength-dependent data compression

The standard GPR framework applies to scalar quantities only. In order to handle multiple quantities (e.g., wavelength-dependent data), one naive approach is to train a separate GPR model for each component. PCA allows reducing the number of components by exploiting redundancy in the data. The model for the *p*-th component is expressed as Ref.^35^

9 $$\begin{aligned} y_p\approx \bar{y}_p+\sum _{n=1}^{\tilde{n}}U_{pn}\mathcal {M}_{\text{GPR},n}(x^*) \end{aligned}$$

where $\bar{y}_p$ is the mean of the training data related to the *p*-th output, $U_{pn}$ is the *p*-th element of the *n*-th singular vector of the training dataset, and $\mathcal {M}_{\text{GPR},n}$ is the GPR model of the *n*-th principal component in the form of (8).

The number of principal components $\tilde{n}$ is selected by setting a relative threshold on the singular values of the training dataset. In this paper, we use PCA to compress wavelength-dependent data related to the same quantity, whereas we apply the whole procedure separately for heterogeneous quantities (i.e. efficiency and average back reflections) and different designs.

## Data Availability

The datasets generated and analyzed in the current study are available from the corresponding author on reasonable request.

## References

[1] Garnett EC, Ehrler B, Polman A, Alarcon-Llado E (2020). Photonics for photovoltaics: Advances and opportunities. ACS Photonics.

[2] Park J (2022). Free-form optimization of nanophotonic devices: From classical methods to deep learning. Nanophotonics.

[3] Ahn GH (2022). Photonic inverse design of on-chip microresonators. ACS Photonics.

[4] Piggott AY (2020). Inverse-designed photonics for semiconductor foundries. ACS Photonics.

[5] Campbell SD (2019). Review of numerical optimization techniques for meta-device design. Opt. Mater. Express.

[6] Zhou M (2021). Inverse design of metasurfaces based on coupled-mode theory and adjoint optimization. ACS Photonics.

[7] Ma W (2021). Deep learning for the design of photonic structures. Nat. Photonics.

[8] Molesky S (2018). Inverse design in nanophotonics. Nat. Photonics.

[9] Melati D (2019). Mapping the global design space of nanophotonic components using machine learning pattern recognition. Nat. Commun..

[10] Dezfouli MK (2020). Perfectly vertical surface grating couplers using subwavelength engineering for increased feature sizes. Opt. Lett..

[11] Wen F, Jiang J, Fan JA (2020). Robust freeform metasurface design based on progressively growing generative networks. ACS Photonics.

[12] Zandehshahvar M (2022). Manifold learning for knowledge discovery and intelligent inverse design of photonic nanostructures: Breaking the geometric complexity. ACS Photonics.

[13] Waqas A, Manfredi P, Melati D (2021). Performance variability analysis of photonic circuits with many correlated parameters. J. Lightwave Technol..

[14] Cheben P, Halir R, Schmid JH, Atwater HA, Smith DR (2018). Subwavelength integrated photonics. Nature.

[15] Xing Y, Spina D, Li A, Dhaene T, Bogaerts W (2016). Stochastic collocation for device-level variability analysis in integrated photonics. Photonics Res..

[16] Xing Y, Dong J, Khan U, Bogaerts W (2022). Capturing the effects of spatial process variations in silicon photonic circuits. ACS Photonics.

[17] Lu Z (2017). Performance prediction for silicon photonics integrated circuits with layout-dependent correlated manufacturing variability. Opt. Express.

[18] Bogaerts W, Xing Y, Khan U (2019). Layout-aware variability analysis, yield prediction, and optimization in photonic integrated circuits. IEEE J. Sel. Top. Quantum Electron..

[19] Manfredi P, Trinchero R (2021). A probabilistic machine learning approach for the uncertainty quantification of electronic circuits based on gaussian process regression. IEEE Trans. Comput. Aided Des. Integr. Circuits Syst..

[20] Kaintura A, Dhaene T, Spina D (2018). Review of polynomial chaos-based methods for uncertainty quantification in modern integrated circuits. Electronics.

[21] Yaghoubi V, Marelli S, Sudret B, Abrahamsson T (2017). Sparse polynomial chaos expansions of frequency response functions using stochastic frequency transformation. Probab. Eng. Mech..

[22] Zhang Z, Batselier K, Liu H, Daniel L, Wong N (2016). Tensor computation: A new framework for high-dimensional problems in eda. IEEE Trans. Comput. Aided Des. Integr. Circuits Syst..

[23] Fuhg JN, Fau A, Nackenhorst U (2021). State-of-the-art and comparative review of adaptive sampling methods for kriging. Arch. Computat. Methods Eng..

[24] Zhou Y, Lu Z (2020). An enhanced kriging surrogate modeling technique for high-dimensional problems. Mech. Syst. Signal Process..

[25] Lee K, Cho H, Lee I (2019). Variable selection using gaussian process regression-based metrics for high-dimensional model approximation with limited data. Struct. Multidiscip. Optim..

[26] Williams CK, Rasmussen CE (2006). Gaussian Processes for Machine Learning.

[27] Kaintura A (2017). A kriging and stochastic collocation ensemble for uncertainty quantification in engineering applications. Eng. Comput..

[28] Gao, Z., Zhang, Z. & Boning, D. S. Few-shot Bayesian performance modeling for silicon photonic devices under process variation. *J. Lightwave Technol.* (2023).

[29] Wang B, Jiang J, Nordin GP (2005). Embedded slanted grating for vertical coupling between fibers and silicon-on-insulator planar waveguides. IEEE Photonics Technol. Lett..

[30] Watanabe T, Ayata M, Koch U, Fedoryshyn Y, Leuthold J (2017). Perpendicular grating coupler based on a blazed antiback-reflection structure. J. Lightwave Technol..

[31] Xu D (2014). Silicon Photonic Integration Platform-Have We Found the Sweet Spot?. IEEE J. Sel. Top. Quantum Electron..

[32] Xing Y, Dong J, Khan U, Bogaerts W (2022). Capturing the effects of spatial process variations in silicon photonic circuits. ACS Photonics.

[33] Dezfouli MK (2020). Perfectly vertical surface grating couplers using subwavelength engineering for increased feature sizes. Opt. Lett..

[34] Dubourg V (2011). Adaptive surrogate models for reliability analysis and reliability-based design optimization.

[35] Manfredi P, Trinchero R (2020). A data compression strategy for the efficient uncertainty quantification of time-domain circuit responses. IEEE Access.

